# A20 Alleviates the Inflammatory Response in Bovine Endometrial Epithelial Cells by Promoting Autophagy

**DOI:** 10.3390/ani14192876

**Published:** 2024-10-06

**Authors:** Junsheng Dong, Bowen Ji, Yeqi Jiang, Fan Fei, Long Guo, Kangjun Liu, Luying Cui, Xia Meng, Jianji Li, Heng Wang

**Affiliations:** 1 College of Veterinary Medicine, Yangzhou University, Jiangsu Co-Innovation Center for Prevention and Control of Important Animal Infectious Diseases and Zoonoses, Yangzhou 225009, China; 17861511563@163.com (B.J.); jiangyq2025@163.com (Y.J.); ffei00127@163.com (F.F.); 18252712741@163.com (L.G.); yzdxlkj@163.com (K.L.); lycui@yzu.edu.cn (L.C.); mengxia_1@126.com (X.M.); jjli@yzu.edu.cn (J.L.); 2 Joint International Research Laboratory of Agriculture and Agri-Product Safety of the Ministry of Education, Yangzhou 225009, China; 3 International Research Laboratory of Prevention and Control of Important Animal infectious Diseases and Zoonotic Diseases of Jiangsu Higher Education Institutions, Yangzhou University, Yangzhou 225009, China

**Keywords:** A20, bovine endometrial epithelial cells, autophagy, inflammatory response, LPS

## Abstract

**Simple Summary:**

Endometritis is a prevalent disease in perinatal dairy cows, which leads to compromised reproductive function. Studies have confirmed that A20, also known as TNFAIP3, can regulate inflammatory responses in various cells. However, it remains unclear whether A20 regulates the inflammatory response of bovine endometrial cells and if autophagy plays a role in A20’s regulation of inflammatory responses. In this paper, we show that elevated expression of A20 is linked to the progression of endometritis in dairy cows. A20 inhibited the activation of the NF-κB pathway and the mRNA expression levels of proinflammatory cytokines in LPS-induced bovine endometrial epithelial cells. Moreover, it enhanced autophagic activity in the bovine endometrial epithelial cells inhibited by LPS. Our study further showed that A20 mitigated the inflammatory response of bovine endometrial epithelial cells by promoting autophagy. These findings will enhance our understanding of the pathogenesis and provide novel insights for the prevention and treatment of endometritis in dairy cows.

**Abstract:**

Endometritis represents a prevalent condition in perinatal dairy cows. Bovine endometrial epithelial cells (BEECs), as the primary interface between cavity and the external environment, are particularly vulnerable to infection by pathogenic bacteria following parturition. A20 is essential for regulating inflammation and modulating immune responses. Nevertheless, the exact role of A20 in the BEECs in response to inflammatory response is not fully understood. An endometritis model infected by *Escherichia coli* (*E. coli*) in vivo and a BEECs inflammation model induced with lipopolysaccharide (LPS) in vitro were built to investigate the function and governing mechanisms of A20 in endometritis. The results showed that infection with *E. coli* resulted in endometrial damage, inflammatory cell infiltration, and upregulation of inflammatory factors in dairy cows. Furthermore, A20 expression was upregulated in the endometrium of cows with endometritis and in BEECs following LPS stimulation. A20 overexpression attenuated the level of proinflammatory cytokines in LPS-stimulated BEECs; conversely, A20 knockdown lead to an exacerbated response to LPS stimulation. The overexpression of A20 was shown to activate autophagy and suppress the NF-κB signaling pathway in LPS-stimulated BEECs. However, blocking autophagy with chloroquine notably attenuated the anti-inflammatory effect of A20, leading to the activation of the NF-κB signaling pathway. In summary, the study demonstrated that A20’s suppression of inflammation in LPS-stimulated BEECs is associated with the activation of autophagy. Therefore, the A20 protein showed potential as a novel treatment focus for managing endometritis in dairy cows.

## 1. Introduction

Bacterial infection, notably, *Escherichia coli* (*E. coli*), commonly leads to postpartum endometritis in dairy cows, resulting in compromised reproductive function [1,2,3]. Lipopolysaccharide (LPS) is a prominent constituent of the Gram-negative bacterial cell envelope and a critical pathogenic factor. Peptidoglycan, a major component of the cell wall of Gram-positive bacteria, induces inflammatory responses that are weaker than those caused by LPS [4]. Upon infection of the endometrium by *E. coli*, substantial quantities of LPS are generated, eliciting an inflammatory response [5,6]. Bovine endometrial epithelial cells (BEECs) serve as the primary barrier by orchestrating immune responses to combat pathogen invasion. Upon LPS stimulation, the inhibitory kappa B (IκB) undergoes phosphorylation and subsequent degradation, facilitating the translocation of nuclear factor kappa B (NF-κB) into the nucleus. Within the nucleus, NF-κB binds to its specific DNA sequences, leading to the activation of downstream inflammatory mediators like tumor necrosis factor-alpha (TNF-α), interleukin-6 (IL-6), and IL-8 [7,8]. The excessive production of these inflammatory cytokines is closely associated with endometritis and may even result in significant damage to the uterus.

A20, an intracellular zinc finger protein, is derived from the tumor necrosis factor-α (TNF-α)-induced protein 3 (TNFAIP3) gene and is essential for regulating the intensity and duration of NF-κB signaling [9,10]. A20 is a recognized anti-inflammatory molecule in the treatment of a range of inflammatory and autoimmune conditions, including inflammatory bowel disease, psoriasis, rheumatoid arthritis, etc. [11,12]. A20 expression is typically low under physiological conditions; however, activated NF-κB signaling transcriptionally upregulates A20 expression by binding to its promoter region in response to pathogenic stimuli [13]. Han et al. demonstrated that minimal expression of A20 was detected in human corneal epithelial cells (HCECs) and normal mouse corneas, but its expression was significantly increased in response to inflammatory stimuli [14]. A20 inhibited the NF-κB signaling and antiapoptotic pathways in gastric epithelial cells upon Helicobacter pylori infection [15]. However, the role of A20 in BEECs stimulated with LPS is not well understood.

As a principal upstream regulator of the inflammatory response, the NF-κB signaling pathway intricately interplays with the autophagy pathway. Increasing evidence supports the role of autophagy in regulating both innate and adaptive immune responses [16,17,18]. Activation of autophagy has been shown to suppress the production of inflammatory cytokines, while impaired autophagy can exacerbate inflammation. In intestinal investigations, impaired autophagy has been shown to lead to inflammation and heighten vulnerability to Crohn’s Disease [19,20]. Hence, targeting autophagy appears to be a promising strategy for modulating and restoring dysregulated inflammatory responses. A previous study has reported that A20 restricted excessive inflammatory responses by inducing autophagy in peripheral blood mononuclear cells with LPS stimulation [21]. However, the interplay between A20, autophagy, and NF-κB in dairy cow endometritis remains unexplored. Therefore, the main objective of this study is to explore the potential of A20 in suppressing inflammatory responses triggered by LPS in BEECs, and to ascertain the potential involvement of autophagy in modulating this regulatory mechanism.

## 2. Materials and Methods

### 2.1. Antibodies and Reagents

Antibodies targeting LC3 (3868), p65 (8242), p-p65 (3033), IκBα (4814), p-IκBα (2859), and β-actin (4970), along with the HRP-conjugated goat anti-rabbit antibody (7074), were sourced from Cell Signaling Technology (Danvers, MA, USA). The antibody targeting A20 (sc-166692) was purchased from Santa Cruz Biotechnology (Dallas, TX, USA). The antibody targeting p62 (18420-1-AP) was procured from Proteintech Group (Wuhan, China). LPS (L2880), DMEM-F12 medium (D8900), and pronase (P5147) were procured from Sigma-Aldrich (St. Louis, MO, USA). Chloroquine (HY-17589A) and Rapamycin (HY-10219) were obtained from MedChemExpress (Shanghai, China). A BCA assay kit (23227), enhanced chemiluminescence kit (34580), and donkey anti-rabbit IgG secondary antibody (A11034) were sourced from Thermo Fisher Scientific (Waltham, MA, USA). A reverse transcription kit (AT341) and an SYBR Green qPCR Super Mix Kit (AQ601) were obtained from TransGen (Beijing, China). TRIzol reagent (DP424) was acquired from Tiangen (Beijing, China).

### 2.2. Animal Treatments

Ten female Chinese Holstein dairy cows were randomly assigned to two groups, each consisting of five cows: the control group and the *E. coli* group. The cows were of similar age, ranging from 18 to 22 months. The individual pens for each treatment group were equipped with unrestricted access to water and feed. *E. coli* was cultured in LB broth under aerobic conditions at 37 °C in a shaking incubator. Subsequently, 10 mL of *E. coli* (10^6^ CFU/mL) suspension in LB broth was infused into the uterine cavity of the cows in the *E. coli* group using a disposable catheter. In contrast, the control group underwent a similar procedure, receiving an infusion of an equal volume of sterile LB broth into the uterine cavity. The dosage of *E. coli* was chosen based on results obtained previously by Socha et al. [22]. After 48 h of infection, cows were slaughtered, and the uterine tissues were collected for histopathological examination and immunohistochemical analysis, as well as to evaluate the expression of inflammatory factors in the endometrium. The Animal Ethical Committee at Yangzhou University evaluated and approved the study procedures for handling and treating animals (202212116).

### 2.3. BEECs Isolation and Treatments

Following established methods, BEECs were obtained and cultured, with the collection and refrigerated transport of uteri from healthy dairy cows to the laboratory [23]. Upon arrival, the serosal layer of the uterus was sterilized using iodophor. The intact endometrium was exposed through longitudinally sectioning the uterine horn, which was then trimmed to a suitable size (approximately 3–4 cm in length) and rinsed with phosphate-buffered saline (PBS). Following 18 h of 0.1% pronase digestion at 4 °C, the tissues were then carefully scraped off the endometrial epithelium using sterile scalpels. The sterile centrifuge tubes containing the collected scrapings were then subjected to centrifugation at 100× *g* for 5 min. The cell precipitate was then resuspended in DMEM/F-12 medium containing 10% fetal bovine serum and 100 U/mL penicillin/streptomycin, after which it was seeded into the appropriate cell culture vessels. The purity of BEECs was assessed through their morphological differences from bovine endometrial stromal cells and immunohistochemical detection of cytokeratin 18 expression [24]. The cells were cultured under standard conditions at 37 °C with 5% CO_2_, with daily medium changes carried out until reaching 90% confluence.

BEECs cultured to the fourth passage were used for subsequent study. Inhibition of autophagy in BEECs was achieved through pre-treatment with chloroquine (CQ, 20μM) for 2 h or interference with the ATG5 gene. BEECs overexpressing or silencing A20 were exposed to LPS (1 μg/mL) for 4 h, with or without autophagy inhibition. The selection of LPS concentration was based on a previous study confirming that 1 μg/mL LPS can represent the concentration of LPS in the uterine lumen of infected cows, as well as our preliminary study [23,24]. A20 overexpressing lentivirus (A20LV), A20 knockdown lentivirus (sh A20), and ATG5 knockdown lentivirus (sh ATG5) were obtained from Tsingke (Beijing, China).

### 2.4. Histological Assay

After being immersed in a 4% formaldehyde solution for 1 week, the endometrial tissues underwent trimming, dehydrating, and were then embedded in paraffin wax. Subsequently, the endometrial tissues underwent sectioning into 4 μm thick slices with a fully automated microtome (Leica RM2255, Leica Biosystems, Wetzlar, Germany) before being placed onto glass slides. To evaluate the histopathological alterations and architectural features the endometrium, staining with Haematoxylin and eosin (HE) was performed.

### 2.5. Immunohistochemistry (IHC) Assay

Following tissue fixation and paraffin embedding, sections were deparaffinized using xylene and rehydrated in water. Subsequently, they were treated with 3% hydrogen peroxide (H_2_O_2_) for 10 min to inhibit the endogenous peroxidase activity, followed by blocking with 5% bovine serum albumin (BSA) for 30 min at 37 °C to minimize non-specific binding. The primary antibodies were then applied and allowed to incubate overnight at 37 °C to facilitate specific antigen-antibody binding. Following this, the sections were exposed to secondary antibodies for 1 h. After immunostaining using 3,3′-diaminobenzidine (DAB), sections were counterstained with hematoxylin to visualize cell nuclei. Finally, the stained sections were observed under a microscope for analysis.

### 2.6. Quantitative Reverse Transcriptase-PCR (qRT-PCR)

The treated BEECs were used for RNA extraction using a TRIzol reagent kit. After reverse transcription of isolated RNA into cDNA, the mRNA expression levels were assessed utilizing a CFX96 Real Time PCR Detection System (Bio-Rad, Hercules, CA, USA) and a SYBR Green qPCR Super Mix Kit. The PCR cycling program started with a 30 s denaturation step at 94 °C, followed by 45 cycles, each consisting of a 5 s denaturation at 94 °C and a 30 s annealing/extension at 60 °C. The endogenous control for normalization in this study was the housekeeping gene β-actin. The relative expression levels of the specific genes were calculated using the 2^−ΔΔCt^ method. The sequences of primers utilized in this study are provided in Table 1 [23,25].

### 2.7. Western Blotting

BEECs were treated with RIPA lysis buffer containing a protease inhibitor for protein extraction. The protein concentration was assessed using the BCA method, and protein samples (30–60 μg) of equal quantities were electrophoresed on 10–12% SDS-PAGE gels, followed by transfer to PVDF membranes. After blocking with a solution of 5% non-fat milk at room temperature for 1 h, specific primary antibodies (A20, LC3, ATG5, p62, p65, p-p65, IκBα, p-IκBα, and β-actin) were incubated with the membranes overnight at 4 °C. Subsequently, secondary antibodies targeting rabbit IgG were applied to the membranes and incubated for 1 h at room temperature. Finally, ImageJ software (ImageJ 1.53q, Bethesda, MD, USA) was utilized to quantify the relative band intensity of the target proteins visualized using an enhanced chemiluminescence (ECL) reagent.

### 2.8. Immunofluorescence Staining

BEECs were seeded onto 24-well plates containing coverslips and cultured in a 5% CO_2_ incubator at 37 °C. After treatment, they were washed three times with PBS, fixed in 4% paraformaldehyde for 15 min and permeabilized with 0.4% Triton X-100 for 10 min. Following blocking with 5% BSA for 1 h at room temperature, the cells were incubated with the primary antibody specific to p65 overnight at 4 °C. After three washes, the cells were treated with Alexa Fluor 594-conjugated donkey anti-rabbit antibody for 1 h. Finally, nuclear staining was performed using DAPI for 5–10 min. The distribution of p65 protein was visualized using laser confocal microscopy. ImageJ 1.53q software was used to quantify and analyze the fluorescence intensity of p65.

### 2.9. Statistical Analyses

Every experiment was conducted in triplicate. All data were expressed as the mean ± standard error of the mean (SEM). The two groups were analyzed statistically using Student’s *t*-test, and multiple groups were analyzed using one-way ANOVA and Tukey’s multiple comparison test. Statistical significance was determined for *p* values below 0.05.

## 3. Results

### 3.1. Inflammatory Damage in the Endometrium of Dairy Cows Was Caused by E. coli

**Figure 1:**
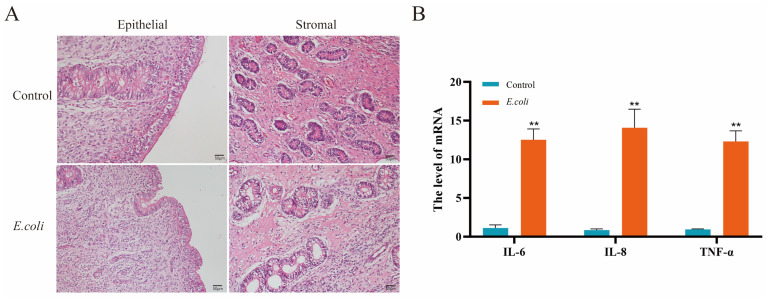
Endometrial inflammatory damage of dairy cow caused by *E. coli*. (**A**) Histopathological changes in endometrium by HE staining. (**B**) The mRNA expression levels of inflammatory factors in bovine endometrium by qRT-PCR. The data represented the mean ± SEM from three separate experiments. ** *p* < 0.01 vs. the control group.

To explore the inflammatory damage caused by *E. coli* in the bovine endometrium, HE staining was utilized to obverse histopathological changes, and qRT-PCR was employed to quantify the levels of inflammatory cytokines expression. As illustrated in Figure 1A, the endometrium appeared structurally intact with no discernible histopathological alterations in the control group. However, in comparison to the control group, *E. coli* infection induced evident damage to the endometrial structure of dairy cows, characterized by epithelial cell disruption, inflammatory cell infiltration, and apparent swelling. As depicted in Figure 1B, the levels of IL-6, IL-8, and TNF-α expression in the endometrium of dairy cows were elevated 48 h post *E. coli* infection. These findings suggested that *E. coli* infection rapidly induced an inflammatory response in the endometrium of dairy cows.

### 3.2. Upregulated Expression of A20 in E. coli-Infected Endometrium and LPS-Stimulated BEECs

**Figure 2:**
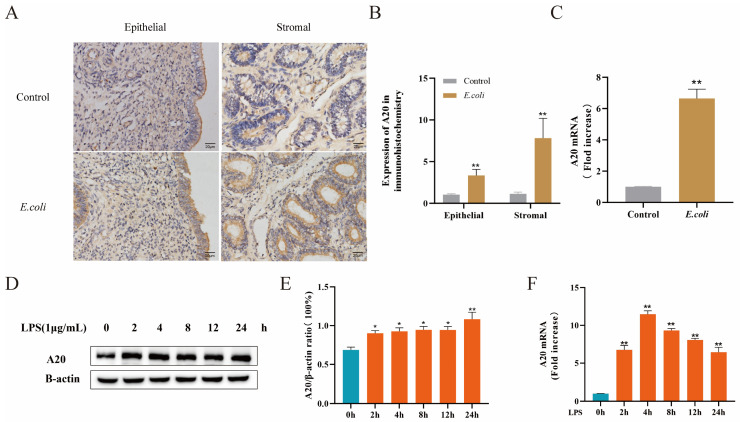
The expression level of A20 in bovine endometrium infected with *E. coli* and LPS-stimulated BEECs. (**A**) The protein expression level of A20 in bovine endometrium by immunohistochemical staining. (**B**) Quantitative analysis of A20 protein expression in bovine endometrium. (**C**) The mRNA expression level of A20 in bovine endometrium by qRT-PCR. (**D**,**F**) The protein and mRNA expression level of A20 following stimulation with 1 μg/mL LPS in BEECs by Western blot and qRT-PCR. (**E**) Quantitative analysis of A20 protein expression in BEECs. The data represent the mean ± SEM from three separate experiments. * *p* < 0.05, ** *p* < 0.01 vs. the control group.

To explore the role of A20 in the endometrium of dairy cows with endometritis, the gene and protein expression levels of A20 in dairy cow endometrium and BEECs were assessed via qRT-PCR, immunohistochemistry, and Western blot analysis. As depicted in Figure 2A–C, there was an increase in A20 protein expression in the endometrium upon infection of the bovine uterus with *E. coli*, as well as an elevation in A20 mRNA expression. The in vitro experimental results were consistent with those in vivo, showing a notable rise in A20 protein and gene expression levels in BEECs induced by LPS from 2 h to 24 h (Figure 2D–F). Therefore, A20 is potentially associated with the inflammatory response of bovine endometrium.

### 3.3. Inhibition of Autophagy Exacerbated Inflammatory Response of BEECs Induced by LPS

**Figure 3:**
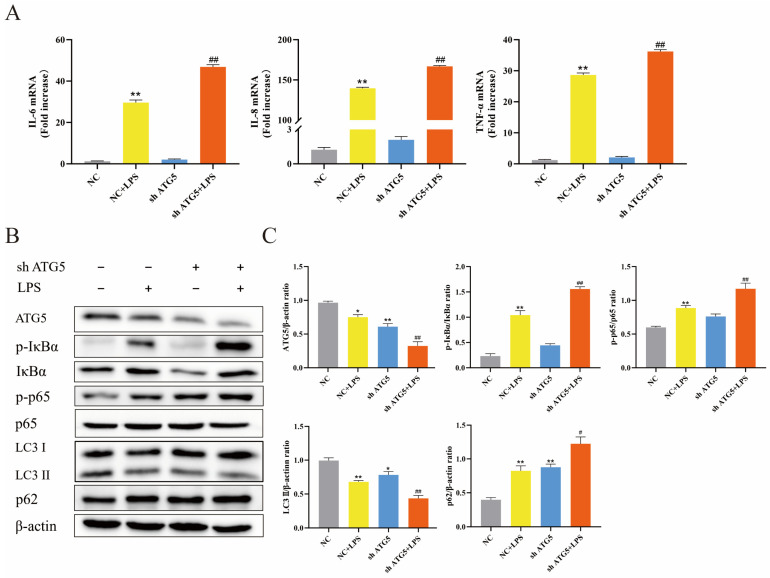
Silencing ATG5 enhanced LPS-induced inflammatory response in BEECs. (**A**) The mRNA expression levels of proinflammatory cytokines in BEECs by qRT-PCR. (**B**) The protein expression levels of ATG5, LC3 II, p62, p-IκBα, and p-p65 in BEECs by Western blot. (**C**) Quantitative analysis of ATG5, LC3 II, p62, p-IκBα, and p-p65. The data represent the mean ± SEM from three separate experiments. * *p* < 0.05, ** *p* < 0.01 vs. the negative control (NC) group, and # *p* < 0.05, ## *p* < 0.01 vs. the NC + LPS group.

To assess the role of autophagy in BEECs during LPS-induced inflammation, lentiviral ATG5 shRNA was transfected into BEECs to inhibit autophagy. As shown in Figure 3B,C, transfection with lentivirus resulted in decreased expression of ATG5 in BEECs, accompanied by reduced levels of LC3 II and increased levels of p62, demonstrating inhibition of autophagic activity in BEECs. Additionally, LPS stimulation alone led to reduced LC3 II levels and elevated p62 levels, indicating that LPS inhibited autophagic activity in BEECs. Figure 3A illustrated the impact of ATG5 knockdown on BEECs stimulated with LPS, resulting in the upregulation of mRNA levels for IL-6, IL-8, and TNF-α. Notably, these pro-inflammatory factors further increased in ATG5-silenced BEECs after LPS stimulation. Additionally, the role of autophagy in the NF-κB signaling pathway was also investigated. As expected, LPS stimulation promoted an increase in the phosphorylation levels of p65 and IκBα. However, following ATG5 knockdown, the phosphorylation levels of p65 and IκBα were further elevated (Figure 3B,C). These findings suggest that suppressing autophagy worsened the inflammation induced by LPS in BEECs.

### 3.4. A20 Inhibited LPS-Induced Inflammatory Responses in BEECs

**Figure 4:**
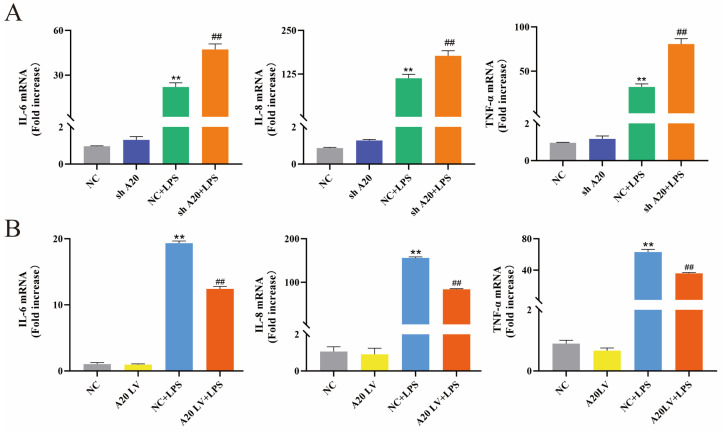
A20 inhibited the inflammatory responses induced by LPS in BEECs. (**A**) The mRNA expression levels of proinflammatory cytokines upon A20 knockdown in BEECs by qRT-PCR. (**B**) The mRNA expression levels of proinflammatory cytokines upon A20 overexpression in BEECs by qRT-PCR. The data represent the mean ± SEM from three separate experiments. ** *p* < 0.01 vs. the NC group, and ## *p* < 0.01 vs. the NC + LPS group.

To investigate the effect of A20 on inflammatory factor expression in BEECs, cells were transfected with lentivirus carrying A20 overexpression or silencing plasmids. The mRNA expression levels of IL-6, IL-8, and TNF-α were evaluated using qRT-PCR. As depicted in Figure 4A, stimulation with LPS increased the expression of IL-6, IL-8, and TNF-α, which could be further aggravated by the silencing of A20. Conversely, as shown in Figure 4B, the overexpression of A20 led to a reduction in the LPS-induced expression of IL-6, IL-8, and TNF-α. Taken together, A20 overexpression attenuated the inflammatory response in BEECs, whereas A20 knockdown exacerbated it.

### 3.5. A20 Inhibited the NF-κB Pathway Activation by Promoting Autophagy

**Figure 5:**
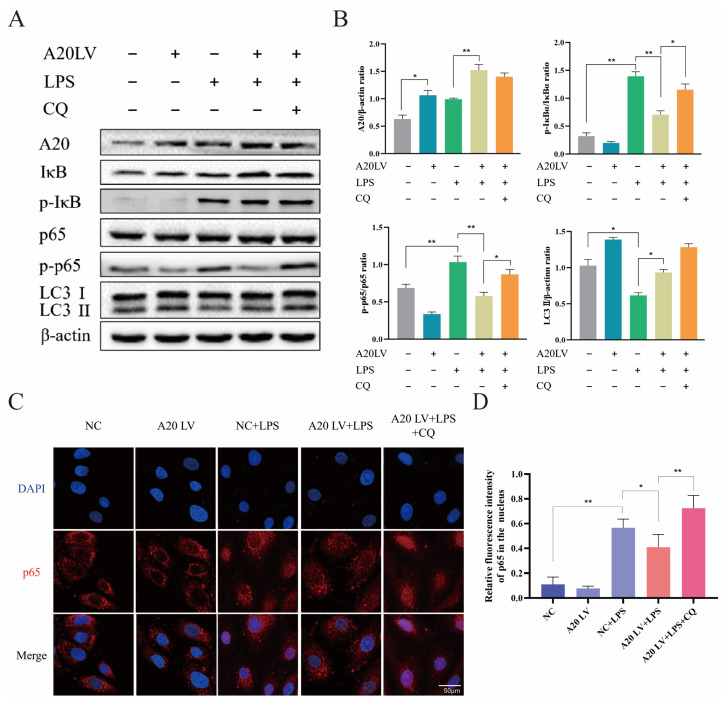
CQ-induced autophagy inhibition attenuated A20-mediated suppression of NF-κB signaling. (**A**) The protein expression levels of A20, p-IκBα, p-p65, and LC3 II by Western blot. (**B**) Quantitative analysis of A20, p-IκBα, p-p65, and LC3 II. (**C**) p65 protein nuclear translocation with immunofluorescence. (**D**) Quantification analysis of nuclear translocation of p65. The data represent the mean ± SEM from three separate experiments. * *p* < 0.05, ** *p* < 0.01.

Figure 3 demonstrated the crucial impact of autophagy on the inflammatory response in BEECs. Subsequently, the involvement of autophagy in mediating the regulatory role of A20 in inflammation was investigated. The efficiency of A20 overexpression was confirmed by Western blot analysis (Figure 5A,B). A20 overexpression reversed autophagy inhibition induced by LPS treatment, resulting in upregulation of LC3 expression, thereby activating autophagy. Following LPS stimulation, A20 overexpression resulted in reduced phosphorylation of p65 and IκBα, indicating its inhibitory effect on NF-κB pathway activation. To explore howA20 regulated the NF-κB pathway through autophagy, the autophagy inhibitor chloroquine (CQ) was used to block autophagy in BEECs. CQ treatment led to elevated phosphorylation levels of p65 and IκBα in the presence of A20 overexpression and LPS stimulation. The immunofluorescence results were similar (Figure 5C,D), showing that CQ treatment significantly increased the nuclear translocation of p65 under the condition of A20 overexpression and LPS stimulation. Taken together, A20 suppressed LPS-induced NF-κB signaling pathway activation in BEECs by upregulating autophagy levels.

## 4. Discussion

Endometritis in dairy cows has deleterious effects on subsequent reproductive performance, including the number of calvings and milk production [26]. Robust uterine defense mechanisms are one of the key factors for the elimination of bacterial infections and postpartum recovery [27]. Therefore, it is crucial to understand the exact pathogenesis of bovine endometritis. In this study, *E. coli* infection induced endometrial damage, inflammatory cell infiltration, and tissue edema in dairy cows, along with a marked upregulation of pro-inflammatory factors. This is consistent with the findings reported by Yang et al., which described endometritis in dairy cows as being associated with the destruction of the endometrial epithelial layer, inflammatory cell infiltration, congestion, stromal edema, and overexpression of inflammatory cytokines [28]. Meanwhile, we observed an upregulation of zinc finger protein A20 expression in the endometrium of dairy cows with endometritis, as well as in LPS-stimulated BEECs. The above results demonstrated that A20 may be involved in regulating the pathogenesis of endometritis in dairy cows.

A20, functioning as a ubiquitin-modifying enzyme with E3 ligase and deubiquitinating properties, serves as a key anti-inflammatory factor in inflammatory and autoimmune diseases, while also being integral to the host’s defense against microbial infections [29,30,31]. High expression of A20 can suppress NF-κB pathway activity by promoting the degradation of key factors in the Toll-like receptor and TNF receptor pathways [32]. A20-null mice displayed severe spontaneous multiorgan inflammation and perinatal mortality [33]. Our findings aligned with previous reports in finding that upon LPS stimulation, A20 overexpression markedly decreased the expression of key pro-inflammatory cytokines, such as IL-6, IL-8, and TNF-α. In contrast, A20 silencing augmented the LPS-induced expression of these cytokines, indicating a pivotal role for A20 in modulating inflammation of BEECs.

Autophagy, a widely preserved cellular mechanism, is crucial for the degradation of impaired organelles and cellular waste within cells [34,35,36]. It is involved in regulating inflammation and restoring homeostasis. Impairment of autophagy, via ATG gene silencing or inhibitors, results in elevated production of pro-inflammatory cytokines. Zhou et al. demonstrated that autophagic dysfunction is linked to intestinal inflammation, with mTOR inhibitors or autophagy activators significantly mitigating LPS-induced colitis in mice and the inflammatory response in intestinal epithelial cells [37]. The study by Liu et al. showed that impaired autophagy is a critical pathogenic factor in the inflammatory damage triggered by *E. coli* in BEECs [38]. Stimulation of autophagic activity effectively mitigated the secretion of pro-inflammatory cytokines, including IL-1β, IL-18, and cytochrome C, consequently alleviating the inflammatory damage caused by *E. coli*. Similarly, this study found that LPS inhibited autophagic activity in BEECs, induced activation of the NF-κB pathway, and elevated the expression levels of inflammatory cytokines IL-8, TNF-α, and IL-6. ATG5, which is crucial for autophagosome formation, was silenced to decrease autophagic activity, which subsequently aggravated the LPS-induced inflammatory response, enhancing NF-κB activation and further increasing inflammatory cytokines levels. Thus, these results indicated that stimulating autophagy can mitigate LPS-induced inflammatory injury in BEECs.

Our findings also revealed that overexpression of A20 enhanced autophagy levels, which were hindered by LPS, concomitantly reducing the levels of p-p65 and p-IκBα, preventing p65 from entering the nucleus and ultimately inhibiting NF-κB pathway activation. Enhanced autophagic activity confers protective effects on innate host defense against bacterial infection [39,40,41]. Further, we inhibited autophagy with inhibitor CQ to explore the role of autophagy in the anti-inflammatory effect of A20. Notably, the inhibitory effect of A20 on the NF-κB pathway was attenuated when autophagy was inhibited. Consistent with our results, Han et al. verified that A20 activated autophagy to inhibit the damage and inflammatory response of corneal epithelial cells and mouse corneas induced by Aspergillus fumigatus [42]. Zhang et al. found that overexpression of A20 attenuated the progression of intervertebral disc degeneration while promoting autophagy in nucleus pulposus cells; conversely, knockout of A20 resulted in reduced levels of autophagy and exacerbated pathology [43]. Therefore, the interaction among A20, autophagy, and the NF-κB pathway is crucial for modulating inflammation. Overall, these results suggest that A20 promoted autophagy activation as a protective response against LPS-triggered inflammatory damage in BEECs.

## 5. Conclusions

In conclusion, this study confirmed that A20 is associated with the progression of bovine endometritis. By inhibiting the activation of the NF-κB pathway and reducing the expression of proinflammatory factors, A20 effectively attenuates the inflammatory response induced by LPS in BEECs. This anti-inflammatory effect may be achieved through the activation of autophagy. These findings will enhance comprehension of the pathogenesis and offer fresh perspectives for the prevention and treatment of endometritis in dairy cows.

## Figures and Tables

**Table 1:** The primer sequences employed for qRT-PCR.

Gene	Primers (5′→3′)	Product Size (bp)	Accession Number
β-actin	F: CATCACCATCGGCAATGAGC R: AGCACCGTGTTGGCGTAGAG	156	NM_173979.3
A20	F: TGCTGCAAAGTTGGATGAAG R: TTGGGACTTTCGTTTGGTTC	281	XM_005210987.2
TNF-α	F: GGGCTTTACCTCATCTACTCACAG R: GATGGCAGACAGGATGTTGACC	132	NM_173966.3
IL-6	F: TGAAAGCAGCAAGGAGACACT R: TGATTGAACCCAGATTGGAAGC	90	NM_173923.2
IL-8	F: TTCCTCAGTAAAGATGCCAATG R: TGACAACCCTACACCAGACCCA	86	NM_173925.2

## Data Availability

The data presented in this study are available in the article.
